# Radiosensitizing properties of dual-functionalized carbon nanostructures loaded with temozolomide

**DOI:** 10.3762/bjnano.16.18

**Published:** 2025-02-19

**Authors:** Radmila Milenkovska, Nikola Geskovski, Dushko Shalabalija, Ljubica Mihailova, Petre Makreski, Dushko Lukarski, Igor Stojkovski, Maja Simonoska Crcarevska, Kristina Mladenovska

**Affiliations:** 1 Institute of Pharmaceutical Technology, Faculty of Pharmacy, Ss. Cyril and Methodius University in Skopje, Blv. Mother Theresa No. 45, 1000 Skopje, Republic of North Macedonia; 2 Institute of Chemistry, Faculty of Natural Sciences and Mathematics, Ss. Cyril and Methodius University in Skopje, Str. Arhimedova No. 5, 1000 Skopje, Republic of North Macedonia; 3 University Clinic of Radiotherapy and Oncology, Faculty of Medicine, Ss. Cyril and Methodius University in Skopje, Blv. Mother Theresa No. 17, 1000 Skopje, Republic of North Macedonia; 4 Faculty of Medicine, Ss. Cyril and Methodius University in Skopje, Str. 50th Division No. 6, 1000 Skopje, Republic of North Macedonia

**Keywords:** carbon nanostructures, cytotoxicity, glioblastoma multiforme, radiosensitizing properties, temozolomide

## Abstract

In the present study, temozolomide (TMZ), a drug used for the treatment of anaplastic astrocytoma and glioblastoma multiforme (GBM), was incorporated into multiwalled carbon nanotubes (MWCNTs) and a MWCNTs–graphene (MWCNTs-G) hybrid compound, covalently functionalized with polyethylene glycol (PEG) 6000 and folic acid (FA), with an aim to prepare nanocarriers with the potential to prolong the drug circulation time, cross the blood–brain–tumor barrier (BBTB), and provide targeted and controlled drug release in the brain tumor cells. Cytotoxicity and effects on cell membrane integrity of the blank and TMZ-loaded dual-functionalized carbon nanostructures (CNs) were evaluated in vitro on a GBM cell line (U87MG), as well as their radiosensitizing properties after exposure of the pre-treated GBM cells to gamma radiation with a standard clinical dose for patients with GBM. All prepared formulations underwent biopharmaceutical and physicochemical characterization, including the formulations exposed to irradiation under the same conditions. For physicochemical characterization of the formulations, different techniques were used by which successful functionalization of the CNs and TMZ loading were confirmed and visualized; no significant changes in the structure of the CNs and TMZ after irradiation were observed. With single and dual functionalization, formulations with relatively high TMZ loading efficiency and drug content were prepared. They exhibited homogeneous particle size distributions and mean particle sizes and surface charges suitable for crossing the BBTB and targeting brain cancer cells. A biphasic drug release profile was observed for all functionalized TMZ-loaded formulations in simulated in vivo conditions, with a sustained release pointing to the potential for controlled release of TMZ in brain tumor cells. The formulations of the hybrid CN MWCNTs-G compared to the corresponding MWCNTs were characterized by a similar or slightly higher TMZ content, larger particle size, similar surface charge, and slightly faster TMZ release, which can be attributed to the planar structure of graphene that promotes TMZ binding to the surface on a larger scale. For the irradiated CNs, lower values for particle size, more positive values for surface charge, and accelerated TMZ release were observed, which could be explained by changes in the physicochemical characteristics of the prepared formulations upon irradiation. Significant concentration-dependent toxicity was observed for blank dual-functionalized CNs, being higher for MWCNTs-G-PEG6000-FA compared to MWCNTs-PEG6000-FA at the same formulation concentrations. With incorporation of TMZ into the functionalized CNs, the cell viability additionally decreased, maintaining the trend for higher cytotoxicity of the hybrid CN. Additional decrease in the viability of cells was observed when GBM cells pre-treated with the corresponding CNs were exposed to irradiation, which could be ascribed to changes in size, surface charge, and release kinetics of TMZ and to irradiation-induced changes in the microenvironment and cell membranes that promote uptake of a larger volume of carriers in the GBM cells. The higher cytotoxicity observed in the hybrid carrier formulations could most likely be attributed to the length of the hybrid carrier and the higher proportion of planar surface, which promotes more intense contact with the cells and rupture of cell membranes. Overall, the findings demonstrate the radiosensitizing properties of not only TMZ but also of CNs and point to a clinical benefit from combined treatment with carbon nanocarriers of TMZ and radiotherapy in GBM.

## Introduction

Carbon-based nanostructures (CNs) such as graphene and its derivatives, carbon nanotubes (CNTs), fullerenes, carbon quantum dots, carbon nanohorns and nanodiamonds (NDs), and their hybrids are becoming a matter of increasing importance in the field of neurology, neuro-oncology, and neuropharmacology. This is because of their specific intrinsic physicochemical, biopharmaceutical, and pharmacological properties such as good bioavailability, relatively low toxicity, low weight and large surface area for active agent loading and functionalization with (intra)cellular component targeting ligands, and extremely small size for crossing the blood–brain barrier (BBB) and targeted delivery to the brain. The hydrophobic nature of the CNs offers good membrane permeability. Through chemical modifications (i.e., oxidation) of their surface, CNs with optimal hydrophobic/hydrophilic properties and increased dispersibility can be obtained as preconditions for biocompatibility and low immunogenicity. Also, improved electronic, mechanical, and thermal properties as preconditions for (photo)thermal and photodynamic therapy can be obtained [1–5]. It has been shown that CNs have an anti-amyloid aggregation activity, and some of them (i.e., carbon nanotubes (CNTs) and graphene) are able to interface with neurons and neuronal circuits and play an important role in the modulation of neurobiological processes, including neuroregeneration, neuronal differentiation, and stimulation of neuronal electrical signalization and brain activity. Thus, they are promising materials for new products regarding tissue engineering and prosthetic neuronal devices [6–8]. There is also an evidence that CNs manifest antiproliferative, antimigratory, and antiangiogenic activities in gliomas due to their direct killing effect through inducing apoptosis and the capability to inhibit genes involved in oxidative phosphorylation and to downregulate genes essential for cytoskeleton formation [9–12]. In addition, an effect on the ectopic (acentrosomal) microtubule nucleation was observed, with disassembly of the centrosome and a cytoskeletal reorganization that trigger the generation of ineffective biomechanical forces, which leads to migration defects, and ultimately to spindle-assembly checkpoint blockage and apoptosis [13]. It was also shown that the CNs impair extracellular adhesion and regulate adhesion-dependent pathways (such as EGFR/AKT/mTOR and β-catenin) [14], inhibit angiogenesis via the NF-κB pathway [15], inhibit several signal transduction pathways (WNT/β-catenin, Notch, and JAK-STAT) [16], promote apoptosis via activation of reactive oxygen species (ROS)-, caspase-, and mitochondrion-dependent pathways, such as p53-mPTP [13,17–18], and reduce the expression of voltage-dependent ion channel genes and extracellular receptors in glioma cells, damaging the cell membrane and changing its potential [19]. These findings are supported by several publications in which CNs were evaluated as potential drug carriers or inherent drugs for brain targeting and (synergistic) treatment of Alzheimer disease [20], Parkinson disease [21], and brain tumors [22–25], and as agents for the detection of brain ischemic stroke [26] and photodynamic and photothermal therapy of brain tumors [27].

Brain tumors are characterized by very high morbidity and mortality rates; among them, the most frequent primary tumors are malignant gliomas, representing more than 80% of all brain tumors, of which more than 40% are glioblastomas. Glioblastoma multiforme (GBM), grade 4 astrocytoma, is the most aggressive and deadly brain tumor, representing 16% of primary brain cancers and up to 54% of all gliomas. The mean survival time estimate for patients with GBM is 14.6 months, the two-year and five-year survival rates are less than 30% and 5%, respectively, while for the patients with unresectable GBM, the prognosis is worse [28–29]. The fast proliferation and aggressive invasion of GBM in the surrounding brain tissue complicate the total resection of the tumor. In addition to the heterogeneity of GBM, the immunosuppressive surrounding, high levels and different mechanisms of drug resistance and radiation tolerance, as well as a blocking effect of the blood–brain–tumor barrier (BBTB) for drug permeation into the brain extracellular matrix lead to high recurrence rates (in up to 90% of patients) even when complete treatment is applied. The standard of care includes surgical resection followed by combined chemo- and radiotherapy (RT), together with non-conventional adjuvant treatment with inhibitors targeting one or a family of biomarkers included in signalization or activation of oncogenic pathways, immunotherapy, gene therapy, and photodynamic therapy.

Cytotoxic drugs acting by preventing DNA replication and apoptosis via methylation of guanine-rich areas in DNA and by cross-linking guanine and cytosine in DNA strains are a standard in chemotherapy. Among them, the prodrug temozolomide (TMZ) with its active metabolite 5-(3-methyl-1-triazene-1-yl)imidazole-4-carboxamide) (MITC) is a first-line therapy for primary and recurrent GBM, especially O^6^-methylguanine-DNA methyltransferase (MGMT)-promotor-methylated tumors. MITC, acting via its metabolite methyl diazonium cation generated at physiological pH (>7), develops O^6^-methylguanine lesions and/or mutations that ultimatively avoid mismatch repair system and lead to apoptosis, autophagia, and cell senescence. Aside from the methylation in the O^6^ position of guanine, TMZ has shown an ability to alkylate other macromolecules with an important role in many cell processes related to changes in the structure of RNA, protein chromatin structure, gene expression and replication, and synthesis and repair of DNA [30]. It has also been shown that TMZ has radiosensitizing effects [31–32], increasing the degradation of DNA strains and cell death when combined with RT, which is a reason for the National Comprehensive Cancer Network to recommend standard RT and adjuvant TMZ plus alternating electric field therapy for patients with GBM at ages up to 70 years with good performance status and having either a “methylated” or “indeterminate” MGMT promoter status [33]. However, the treatment with TMZ is associated with several disadvantages such as excessive toxicity for the surrounding tissues, short half-life of 1.8 h (which requires frequent administrations for achieving therapeutic concentrations at the site of action and leads to unfavorable safety profile and tolerability), and limited efficacy (due to the high level of DNA repair by MGMT and other mechanisms of resistance) [34]. In addition, the high hydrolytic instability of TMZ at pH ≥ 7 limits its efficacy, because the formed MTIC exhibits high systemic toxicity and cannot pass the BBTB and accumulate in the GBM, unlike TMZ. Therefore, it is of paramount importance to prolong the half-life of TMZ at physiological pH and to increase its accumulation in the tumor cells prior to degradation in the central circulation.

Several strategies have been exploited to stabilize TMZ and overcome the disadvantages. Among them is the incorporation of TMZ in organic and inorganic nanomaterials and their hybrids, designed in a wide variety of shapes such as nanoparticles (NPs), conjugates, dendrimers, and liposomes [35]. With various bioengineering techniques, the nanomaterials’ size, shape, and surface properties were modified to improve formulation, biopharmaceutics/release kinetics, and pharmacokinetics of TMZ. Also, surface functionalization attempts with multiple targeting ligands were made to deliver TMZ to the site of interest, exploiting the site-specific expression or overexpression of specific molecules on BBTB and GBM cells to enable transport and delivery to brain tumors. Inorganic nanostructures as TMZ carriers have shown several advantages compared to organic ones with respect to physicochemical stability and potency/cytotoxic activity, overcoming their main disadvantages, that is, hydrophobicity/fluidity and toxicity by chemical modifications and/or coupling with hydrophilic polymers. TMZ was successfully incorporated in magnetic NPs [36], mesoporous silica NPs [37], and NPs made of silver [38], zinc oxide [39], and gold [40], all of them showing high accumulation in tumor cells and cytotoxic activity in vitro and in vivo.

Despite the beneficial properties, CNs have not been extensively researched as inherent drugs or drug/TMZ carriers for the treatment of GBM. In two papers, a hybrid made of carbon quantum dots functionalized with chitosan, polyethylene oxide, and carboxymethyl cellulose–polyvinyl alcohol provided controlled release of TMZ [41–42]. In another publication [25], the suitability of graphene oxide (GO) functionalized with folic acid (FA) for controlled release of TMZ and the inhibition of glioma growth was confirmed in vivo. To our knowledge (and stated also in the paper of Petrenko et al. [35]), our group was the first one that incorporated TMZ in multiwalled CNTs (MWCNTs), and their hybrid with graphene (MWCNT-G), non-covalently functionalized with polyethylene glycol (PEG), and pointed to their suitability for targeted and controlled brain delivery, based on their physicochemical and biopharmaceutical properties [43]. In this paper, TMZ was incorporated in the same carriers, this time covalently dual-functionalized with PEG (average *M*_w_ 6000 g/mol) and FA, with an overall aim to prolong the TMZ circulation time (through the stealth effect of PEG) and to increase permeability through the BBTB and accumulation in the brain tumor cells (through the active targeting of the formulations towards membrane folate receptors of GBM). Detailed physicochemical and biopharmaceutical characterizations of the prepared TMZ-loaded dual-functionalized CNs was performed, and the cytotoxicity/radiosensitizing properties of the functionalized CNs with and without TMZ were investigated and compared in vitro, using human glioblastoma cell line exposed to irradiation with a dose rate used in clinical settings for most of the patients with GBM. Simultaneously, the formulations exposed to irradiation under the same conditions were characterized in terms of their physicochemical and biopharmaceutical properties.

## Results and Discussion

### Biopharmaceutical characterization of temozolomide-loaded carbon nanostructures

#### Loading efficacy, drug content, surface charge, and particle size distribution

In the study, relatively high values for TMZ loading efficacy and content were achieved, ranging from 42% to 67% and from 11% to 18%, respectively (Table 1), which are in the scope of values reported for nanocarriers of TMZ (27% to 89% and 4% to 11%, respectively) [44–46]. The higher values achieved for plain (non-functionalized) formulations relative to the functionalized ones (Table 1) could be explained by the formation of strong covalent bonds between CNs and PEG6000, the competition between PEG6000 and TMZ regarding interactions with MWCNTs-COOH and MWCNTs-G-COOH, and the physical entrapment of TMZ in the tubes. One can assume that TMZ is both physically entrapped in the tubes and wrapped around the CNs because of electrostatic and hydrogen bond interactions with CNs and PEG6000. This assumption is supported by the similar values for loading efficacy and drug content of covalently PEGylated TMZ-loaded MWCNTs and hybrid MWCNTs-G (Table 1), which differ in the fraction of tubes. When analyzing the data for dual-functionalized formulations with TMZ, one can see that FA acts like an additional competitor for drug loading (although interactions between TMZ and FA cannot be excluded), indicated by the lower values for loading efficacy and content in these formulations (around 42% and 11%, respectively, for MWCNTs-PEG6000-FA-TMZ and 46% and 13%, respectively, for MWCNTs-G-PEG6000-FA-TMZ; Table 1).

**Table 1:** Loading efficacy, drug content, size distribution, and surface charge of TMZ-loaded carbon nanostructures.

Series	Parameters			

	loading efficacy (±SD, *n* = 6) %	drug content (±SD, *n* = 6) % (theor. 25%)	*d*_50_/PDI (nm)	zeta potential (±SD, *n* = 6) mV

MWCNTs-COOH^a^	—	—	136/0.460	−38.38 ± 0.85
MWCNTs-PEG6000^b^	—	—	231/0.383	−21.40 ± 0.93
MWCNTs-PEG6000-FA^c^	—	—	270/0.364	−33.10 ± 0.60
I-MWCNTs-PEG6000-FA^d^	—	—	222/0.340	−21.05 ± 1.72
MWCNTs-TMZ^e^	66.96 ± 0.60	17.94 ± 0.60	163/0.385	−27.45 ± 1.37
MWCNTs-PEG6000-TMZ^f^	48.68 ± 1.84	14.53 ± 1.61	282/0.402	−25.40 ± 0.55
MWCNTs-PEG6000-FA-TMZ^g^	41.92 ± 4.12	11.04 ± 0.76	300/0.495	−30.70 ± 0.32
I-MWCNTs-PEG6000-FA-TMZ^h^	41.92 ± 4.12	11.04 ± 0.76	244/0.307	−19.87 ± 1.35
MWCNTs-G-COOH^i^	—	—	222/0.406	−46.05 ± 1.15
MWCNTs-G-PEG6000^j^	—	—	322/0.442	−22.30 ± 1.10
MWCNTs-G-PEG6000-FA^k^	—	—	374/0.541	−38.10 ± 0.82
I-MWCNTs-G-PEG6000-FA^l^	—	—	305/0.327	−18.57 ± 1.15
MWCNTs-G-TMZ^m^	58.20 ± 1.60	16.11 ± 0.45	255/0.479	−31.23 ± 2.02
MWCNTs-G-PEG6000-TMZ^n^	49.40 ± 4.42	14.56 ± 1.22	388/0.531	−25.00 ± 0.70
MWCNTs-G-PEG6000-FA-TMZ^o^	46.13 ± 3.10	12.67 ± 2.15	415/0.386	−35.89 ± 0.91
I-MWCNTs-G-PEG6000-FA-TMZ^p^	46.13 ± 3.10	12.67 ± 2.15	347/0.218	−16.50 ± 1.03

^a^MWCNTs-COOH (oxidized MWCNTs); ^b^MWCNTs-PEG6000 (MWCNTs covalently functionalized with PEG6000); ^c^MWCNTs-PEG6000-FA (MWCNTs dual-functionalized with PEG6000 and FA); ^d^I-MWCNTs-PEG6000-FA (irradiated MWCNTs dual-functionalized with PEG6000 and FA); ^e^MWCNTs-TMZ (temozolomide-loaded MWCNTs); ^f^MWCNTs-PEG6000-TMZ (temozolomide-loaded MWCNTs covalently functionalized with PEG6000); ^g^MWCNTs-PEG6000-FA-TMZ (temozolomide-loaded MWCNTs dual-functionalized with PEG6000 and FA); ^h^I-MWCNTs-PEG6000-FA-TMZ (irradiated temozolomide-loaded MWCNTs dual-functionalized with PEG6000 and FA); ^i^MWCNTs-G-COOH (oxidized MWCNTs/graphene hybrid); ^j^MWCNTs-G-PEG6000 (MWCNTs/graphene hybrid covalently functionalized with PEG6000); ^k^MWCNTs-G-PEG6000-FA (MWCNTs/graphene hybrid dual-functionalized with PEG6000 and FA); ^l^I-MWCNTs-G-PEG6000-FA (irradiated MWCNTs/graphene hybrid dual-functionalized with PEG6000 and FA); ^m^MWCNTs-G-TMZ (temozolomide-loaded MWCNTs/graphene hybrid); ^n^MWCNTs-G-PEG6000-TMZ (temozolomide-loaded MWCNTs/graphene hybrid covalently functionalized with PEG6000); ^o^MWCNTs-G-PEG6000-FA-TMZ (temozolomide-loaded MWCNTs/graphene hybrid dual-functionalized with PEG6000 and FA); ^p^I-MWCNTs-G-PEG6000-FA-TMZ (irradiated temozolomide-loaded MWCNTs/graphene hybrid dual-functionalized with PEG6000 and FA).

When the CNs were PEGylated, an increase in zeta potential was observed (−38.38 and −46.05 mV vs −21.40 and −22.30 mV for MWCNTs-PEG6000 and MWCNTs-G-PEG6000, respectively) (Table 1) due to modifications of the carboxylic groups on the surface of CNs. The additional functionalization with FA decreased the zeta potential (−33.10 and −38.10 mV for MWCNTs-PEG6000-FA and MWCNTs-G-PEG6000-FA, respectively), which can be ascribed to the ionized carboxyl groups of FA in the corresponding medium. Incorporation of TMZ in the CNs did not change their zeta potential significantly, confirming the assumptions regarding the TMZ loading (Table 1).

The mean particle size for the parent CNs (MWCNTs-COOH and MWCNTs-G-COOH) was 136 and 222 nm, respectively (Table 1). The most significant changes in the particle size was observed after covalent PEGylation (increase to 231 and 322 nm, respectively). The larger mean particle size observed for covalently modified CNs vs non-covalently modified ones supports the assumption of a higher content of polymer on the surface of the covalently modified CNs (observed also with thermogravimetric analysis (TGA)). After coupling with FA, an additional increase in the mean particle size was observed, with values of 270 and 305 nm for MWCNTs-PEG6000-FA and MWCNTs-G-PEG6000-FA, respectively. This points to surface attachment of FA, although its physical entrapment into the tubes cannot be excluded. After incorporation of TMZ, the mean particle size was increased by 30–40 nm, suggesting again the embedment of TMZ not only into the tubes, but also on the surface of the CNs (as shown in the SEM images in this study and the SEM and TEM images in [43]). In all series, a relatively unimodal particle size distribution was observed, with PDI values not higher than 0.541.

In the irradiated series, the mean particle size ranged from 222 nm (I-MWCNTs-PEG6000-FA) to 347 nm (I-MWCNTs-G-PEG6000-FA-TMZ) (Table 1). Generally, the particle size was smaller compared to the size of the corresponding non-irradiated formulations, which can be attributed to the penetration power and destructive effect of the gamma rays and the dissociation of certain molecules from the nanocarriers into the surrounding medium. Such a trend was observed in a study of Jun et al. [47] in which MWCNTs conjugated with chitosan oligomers and with incorporated tea polyphenols for cancer treatment were irradiated by gamma rays from ^60^Co for 30 min with a dose of 1.5 Gy. The irradiation also led to changes in zeta potential to lower values (i.e., more positive than those of non-irradiated series), ranging from −21.05 mV (I-MWCNTs-PEG6000-FA) to −16.50 mV (I-MWCNTs-G-PEG6000-FA-TMZ) (Table 1).

In general, in the present study, carbon-based TMZ carriers with suitable surface charge for the established aim were prepared, considering that particles with negative (and neutral) zeta potential have a higher capability to escape opsonization and accumulation in liver and spleen (which is a precondition for prolonged circulation time of the particles/TMZ). In addition, particles with suitable size (between 50 and 400 nm) for permeation through the BBTB and uptake into the tumor cells were obtained, as a precondition for higher extent and rate of their internalization and optimal risk/benefit ratio of the treatment with TMZ. This knowledge is based on a lot of publications in which nanoparticulated formulations of different materials designed for brain delivery, including carbon-based, with moderately negative (between −1 and −15 mV) or highly negative zeta potentials (between −15 and −45 mV) and particle sizes between 100 and 400 nm, showed the capability to cross the BBTB and accumulate in the brain tumor cells in vitro and in vivo [48–53].

#### Morphology

**Figure 1:**
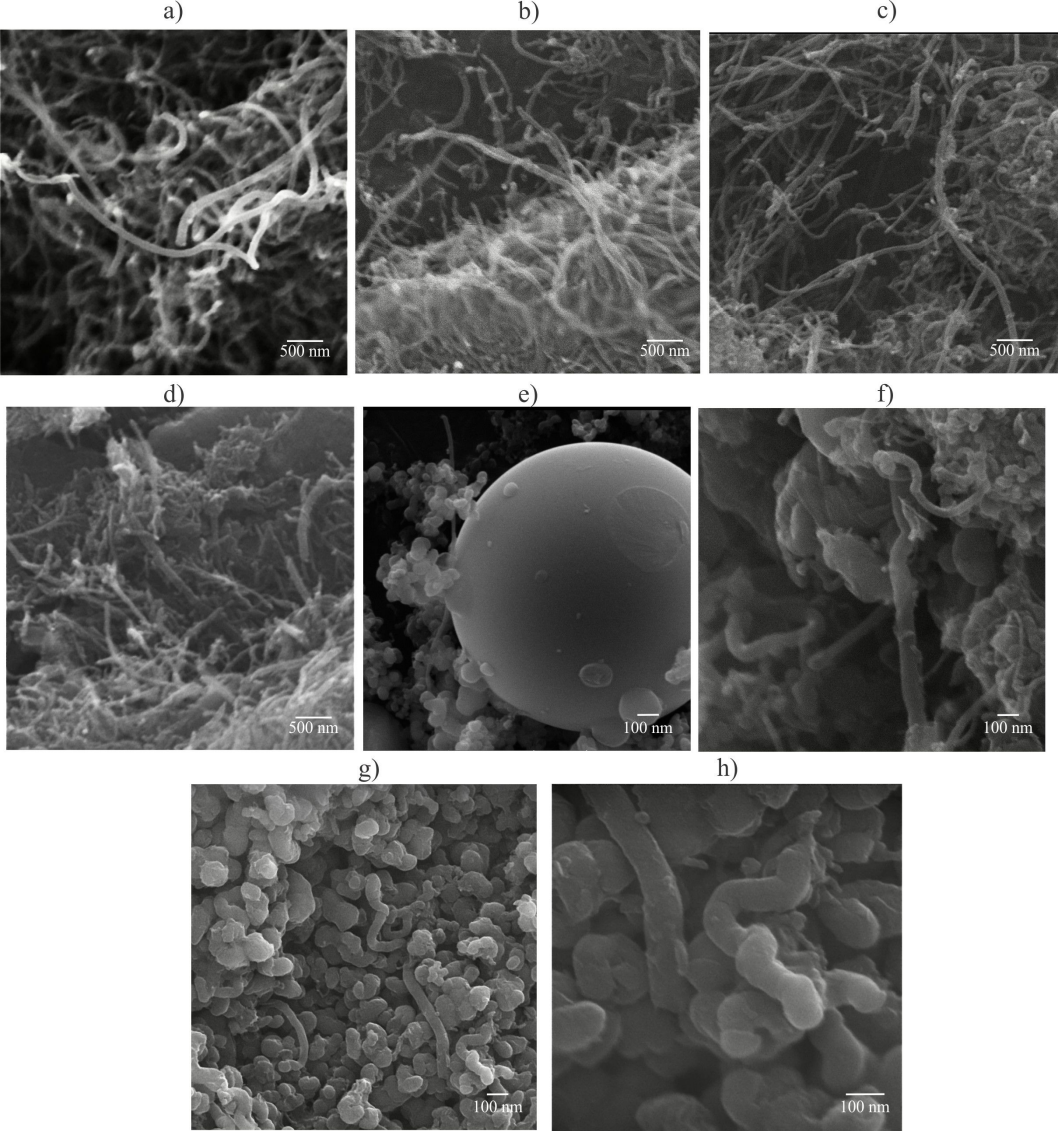
SEM images of (a) MWCNTs-PEG6000, (b) MWCNTs-PEG6000-FA, (c) MWCNTs-PEG6000-TMZ, (d) MWCNTs-PEG6000-FA-TMZ, (e) MWCNTs-G-PEG6000, (f) MWCNTs-G-PEG6000-FA, (g) MWCNTs-G-PEG6000-TMZ, and (h) MWCNTs-G-PEG6000-FA-TMZ.

In the SEM images of the MWCNTs-COOH (presented in our previous study [43]), a dense structure of randomly aggregated, convoluted, and highly tangled tubes was observed. The image of MWCNTs-G shows a hybrid structure of multiwalled nanotubes dispersed within the graphene sheets, formed by interactions between the hydrophobic regions of graphene and the side walls of MWCNTs. Also, in the SEM and TEM images of TMZ-loaded non-modified CNs, entrapment of TMZ into the tubes and wrapping around the CNs was visible [43]. In the present study, the covalent PEGylation and dual functionalization of MWCNTs (Figure 1a–d) was visible by enlarged tubes/thicker walls and non-uniform surfaces of the tubes. The images of PEGylated and dual-functionalized hybrid MWCNTs-G (Figure 1e–h) showed, in addition to the thicker side walls and rounded ends of the tubes, spherical structures attached to the graphene sheets, attributed dominantly to PEG6000. No clear distinction regarding the morphology of single- and dual-functionalized CNs and structures with and without TMZ could be made with the used imaging technique. Also, no morphological differences were observed between the irradiated and non-irradiated CNs.

#### In vitro release

**Figure 2:**
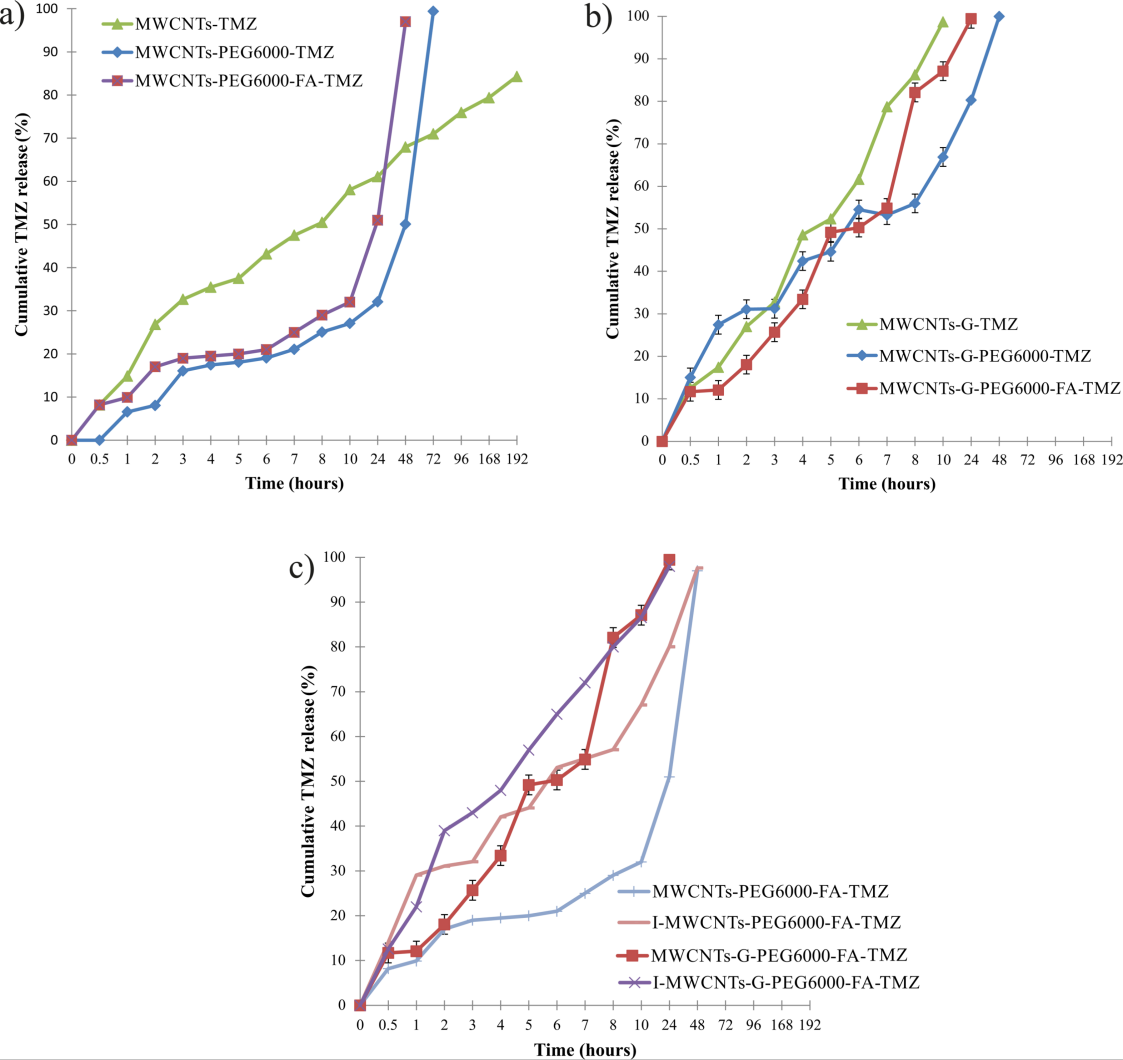
Cumulative release of TMZ from (а) plain, covalently PEGylated, and additionally FA modified MWCNTs, (b) plain, covalently PEGylated, and additionally FA modified MWCNTs-G, and (c) non-irradiated and irradiated MWCNTs and MWCNTs-G covalently PEGylated and modified with FA.

All functionalized CNs loaded with TMZ manifested a biphasic release profile, with an initial burst release and a phase of continuous release (observed also in non-covalently PEGylated formulations in our previous study [43]). In the initial phase of 2 h, 16% and 27% of the loaded TMZ were released from MWCNTs-PEG6000-TMZ and MWCNTs-G-PEG6000-TMZ formulations, respectively (Figure 2a,b), which can be attributed to the release of TMZ located on the surface of the CNs. A lower percentage of TMZ was initially released from the covalently PEGylated CNs compared to the non-covalently PEGylated ones prepared in our previous study [43] (35% and 41%, respectively). This can be explained by the higher content of PEG6000 in the covalently functionalized formulations (confirmed by TGA also) and its barrier role in the TMZ release from the CNs. However, when considering the whole process of drug release, faster release was observed from covalently PEGylated formulations compared to non-covalently PEGylated formulations. Namely, the total content of TMZ loaded in MWCNTs-PEG6000-TMZ and MWCNTs-G-PEG6000-TMZ was released after 72 h (Figure 2a) and 48 h (Figure 2b), respectively, in contrast to non-covalently PEGylated formulations from which the complete release of TMZ occurred over a period of 192 h [43]. The reason is the lower content of TMZ entrapped into the tubes, that is, the higher content of surface-bound TMZ in covalently PEGylated formulations. This can also explain the difference in drug release between the covalently PEGylated and non-PEGylated MWCNTs; over a period of 72 h, around 71% of the drug was released from MWCNTs-TMZ and 99% from MWCNTs-PEG6000-TMZ (Figure 2a). The same trend was observed for the dual-functionalized formulations, MWCNTs-PEG6000-FA-TMZ and MWCNTs-G-PEG6000-FA-TMZ, from which initially similar or slightly lower quantities of TMZ were released (17% and 18%, respectively) compared to the covalently PEGylated (and non-covalently PEGylated) formulations, but from which the total TMZ content was released faster, that is, after 48 h. One can suppose that this release profile can be also attributed to the additional barrier (consisting of PEG6000 and FA) for the entrapment of TMZ into the tubes. When comparing the drug release profiles of the covalently PEGylated MWCNTs and MWCNTs-G formulations, whether modified with FA or not, one can see a faster release from the hybrid structure, which can be again explained by lower TMZ content in the tubes and higher content of surface-bound TMZ in the hybrid structure and the resulting faster diffusion in the dissolution medium. The higher content of surface-bound TMZ in the non-modified hybrid structure could also explain the faster TMZ release from this formulation in comparison with the functionalized ones (Figure 2b).

After irradiation, the TMZ release from the dual-functionalized CNs was slightly faster, which can be attributed to the changes in the size of the CNs, faster dissociation of TMZ into the surrounding medium, and structural changes of the CNs caused by irradiation. In the initial 2 h, 30% and 40% of TMZ from I-MWCNTs-PEG6000-FA-TMZ and I-MWCNTs-G-PEG6000-FA-TMZ, respectively, were released, followed by complete release after 24 and 48 h, respectively (Figure 2c).

Carbon-based TMZ carriers with a favorable release profile have been prepared considering the importance of controlled and sustained drug release to prevent fast TMZ degradation, postsurgical sensitization on radiotherapy, less frequent drug administration, and improved risk/benefit ratio in patients with malignant (recurrent) glioma. These findings are supported by several studies in which the controlled release of TMZ was provided by loading in nanoparticulated carriers, with subsequent improved brain uptake, increased potency, and lower systemic toxicity [54–59]. Controlled release was also provided through loading TMZ in a hybrid compound of carbon quantum dots, chitosan, polyethylene oxide, and carboxymethyl cellulose–polyvinyl alcohol (CS-PEO-CQDs/CMC-PVA) via coaxial spinning, and a transport system of CMC-PVA coating and CS-PEO-CQDs core was formed [41–42]. In a study of Wang et al. [25], formulations made of TMZ-loaded and FA-functionalized GO provided pH-dependent and controlled TMZ release, and the favorable release profile was further confirmed in vivo by successful inhibition of glioma growth. In our previous study [43], in which non-covalent PEGylation of the same TMZ-loaded CNs was performed using PEG1500, PEG4000, and PEG6000, sustained release over extended periods of time was also observed, with no significant difference in the drug release profile between the different PEGylated formulations. Therefore, in the current study, PEG6000 was used for covalent functionalization, based on the assumption that the polymer with the higher molecular weight would provide longer circulation time and, thus, higher uptake ratio in the brain tumor cells.

### Physicochemical characterization of temozolomide-loaded carbon nanostructures

For physicochemical characterization of all formulations regarding interactions of the components and their stability during the preparation procedures, different techniques were used including infrared (IR), ultraviolet–visible (UV–vis), and Raman spectroscopy as well as TGA. For analyzing potential structural changes in the CNs after exposure to irradiation, X-ray powder diffraction (XRPD) was used. The stability of TMZ under these conditions was determined unsing attenuated total reflectance Fourier-transform infrared (ATR-FTIR) and UV–vis spectroscopy. Most of the procedures and techniques were used in our previous study [43] in which physicochemical properties of non-covalently PEGylated CNs loaded with TMZ were characterized.

#### Ultraviolet-visible absorption spectroscopy

**Figure 3:**
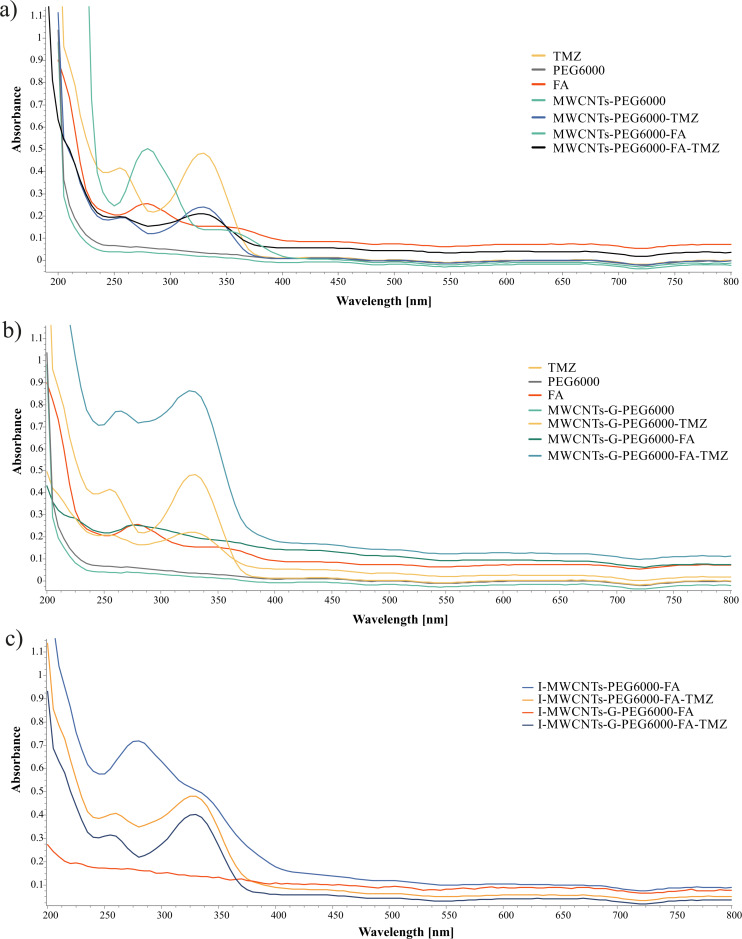
UV–vis spectra of (а) TMZ, PEG6000, FA, and blank and TMZ-loaded single- and dual-functionalized MWCNTs; (b) TMZ, PEG6000, FA, and blank and TMZ-loaded single- and dual-functionalized MWCNTs-G; and (c) irradiated blank and TMZ-loaded dual-functionalized CNs.

UV–vis absorption spectroscopy turned out to be a useful tool for characterizing functionalization with FA and for confirming the TMZ loading through the characteristic absorption peaks of FA (at 280 nm) and TMZ (at 255 and 328 nm, corresponding to the active hydrolytic metabolite MTIC and the prodrug TMZ, respectively) in water solution and the concurrent absence of peaks in water solution of free PEG6000 and unloaded non-PEGylated and PEGylated CNs (Figure 3).

In the UV–vis spectra of TMZ-loaded covalently PEGylated CNs, the two characteristic peaks of TMZ were present, as well as in the spectra of MWCNTs-PEG6000-FA-TMZ (Figure 3a) and MWCNT-G-PEG6000-FA-TMZ (Figure 3b), in which the characteristic peak of FA was also visible. No difference between the UV–vis spectra of irradiated and non-irradiated formulations was observed, and no changes in the spectra of TMZ released from I-MWCNTs-PEG6000-FA-TMZ and I-MWCNTs-G-PEG6000-FA-TMZ were observed when comparing with the spectra of TMZ released from the corresponding non-irradiated formulations.

#### Infrared spectroscopy

**Figure 4:**
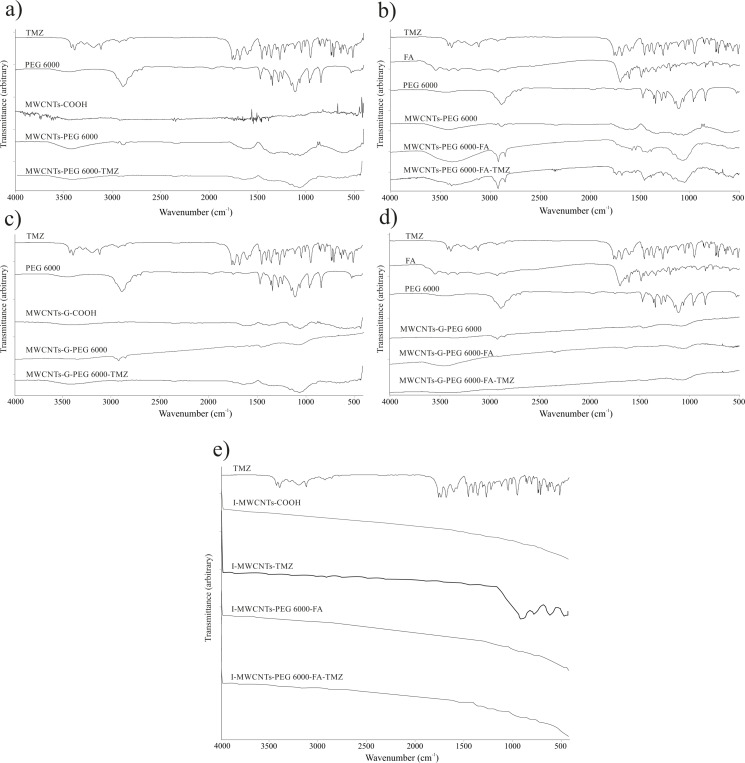
Comparison of IR spectra of individual components and blank and TMZ-loaded (a) PEGylated MWCNTs, (b) dual-functionalized MWCNTs, (c) PEGylated МWCNTs-G, and (d) dual-functionalized MWCNTs-G. (е) Comparison of ATR-FTIR spectra of TMZ and irradiated blank and TMZ-loaded MWCNTs-COOH and dual-functionalized MWCNTs-COOH.

IR spectroscopy was used to analyze the presence of different functional groups in the CNs and interactions formed during single and dual functionalization and TMZ loading. The IR spectra of PEG6000, MWCNTs-COOH, MWCNTs-G-COOH, and TMZ were already characterized in our previous study [43], and they are also presented in Figure 4.

When analyzing the IR spectra of PEG6000, oxidized MWCNTs-COOH, and covalently PEGylated MWCNTs-PEG6000 (Figure 4a), one can see that upon covalent functionalization, the spectrum of MWCNTs-COOH significantly changed. Namely, the bands at 3423 and 3390 cm^−1^ in the spectrum of MCWNTs-COOH, assigned to O–H stretching vibrations, were significantly more intense in the spectrum of MWCNTs-PEG6000. Bands at 2917 and 2852 cm^−1^ emerged in the spectrum of MCWNTs-PEG6000, which are attributed to asymmetric and symmetric C–H stretching vibrations of the CH_2_ groups in PEG6000. The band at 1730 cm^−1^ (originating from C=O stretching vibrations) in the spectrum of MWCNTs-COOH has a lower intensity in the MWCNTs-PEG6000 spectrum as a result of interaction between MWCNTs-COOH and PEG6000. In addition, a new band evolved in the spectrum of MWCNTs-PEG6000 at 1048 cm^−1^ (attributed to stretching vibrations of C–O bonds in PEG6000), which disappeared in the spectrum of MWCNTs-COOH. In summary, the changes in the spectrum of MWCNTs-PEG6000 relative to the spectrum of MWCNTs-COOH indicate the successful covalent PEGylation of MWCNTs. The covalent PEGylation was also evident in the spectrum of MWCNTs-G-PEG6000 (Figure 4c) by the bands between 2950 and 2850 cm^−1^, originating from C–H stretching vibrations of PEG6000, which are absent in the spectrum of MWCNTs-G-COOH. Furthermore, the characteristic band at 1467 cm^−1^ found in the spectra of both PEG6000 and MWCNTs-G-PEG6000 is assigned to CH_2_ deformation vibrations. A characteristic band also occurred at 1078 cm^−1^, which can be assigned to asymmetric C–O–C stretching vibrations in PEG6000. The new band in the spectra of MWCNTs-PEG6000 and MWCNTs-G-PEG6000 at 1730 cm^−1^, which is shifted from 1703 cm^−1^ in the spectra of MWCNTs-COOH and MWCNTs-G-COOH (assigned to C=O vibrations from the carboxyl groups of PEG6000) probably implies on ester bond and confirms the covalent PEGylation of the CNs.

Upon FA functionalization, the spectra of MWCNTs-PEG6000-FA (Figure 4b) and MWCNTs-G-PEG6000-FA (Figure 4d) showed a new band at 1679 cm^−1^, probably from C=O stretching vibrations within the FA molecular structure. The spectrum of FA exhibits a band at 1639 cm^−1^ from the amide C=O group, which is shifted to 1604 and 1637 cm^−1^ in the spectra of MWCNTs-PEG6000-FA and MWCNTs-G-PEG6000-FA, respectively. The spectra of MWCNTs-PEG6000-FA and MWCNTs-G-PEG6000-FA exhibit another novel band at 1728 and 1732 cm^−1^, respectively, resulting probably from C=O stretching vibrations of the ester RCOOR′ group formed between the carboxyl groups of FA and hydroxy groups of PEG6000. The bands at 1423 cm^−1^ in the spectrum of MWCNTs-PEG6000-FA and at 1428 cm^−1^ in the spectrum of MWCNTs-G-PEG6000-FA correspond to deformation vibrations of C–H bonds in the aromatic ring of FA, pointing to a successful functionalization of the PEGylated CNs with FA.

The IR spectrum of TMZ (presented also in our previous work [43]) depicts three wide bands at 3339, 3381, and 3426 cm^−1^, attributed to the NH_2_ and OH stretching vibrations, as well as two bands at 2927 and 2855 cm^−1^, related to asymmetric and symmetric stretching vibrations of aliphatic methylene groups. All these bands are stronger in the spectra of MWCNTs-PEG6000-FA-TMZ (Figure 4b) and MWCNTs-G-PEG6000-FA-TMZ (Figure 4d), but are slightly shifted to 3346, 3389, 3423, 2921, and 2853 cm^−1^, serving as a spectroscopic evidence for the existence of non-covalent interactions (electrostatic, hydrogen bond, and/or van der Waals forces) between CNs and TMZ.

From comparison of the ATR-FTIR spectrum of TMZ with the spectra of the irradiated formulations I-MWCNTs-COOH, I-MWCNTs-TMZ, I-MWCNTs-PEG6000-FA, and I-MWCNTs-PEG6000-FA-TMZ (Figure 4e,f), it becomes obvious that the specific bands of TMZ appear in the spectra of the drug-loaded formulations. The most evident features are the bands at 1106 and 1046 cm^−1^, resulting from C–H deformation vibrations in the aromatic ring of TMZ, as well as the band at 699 cm^−1^, originating either from rocking vibrations of PEG6000 or C–H out-of-plane deformation vibrations in TMZ. The appearance of bands in the spectrum of TMZ and their absence in the spectra of blank CNs indicate that the structure of TMZ was preserved during irradiation. Upon its incorporation in the CNs, in vitro dissolution studies, in which UV–vis spectra of TMZ after its release from the irradiated CNs were recorded, were carried out.

#### Raman spectroscopy

**Figure 5:**
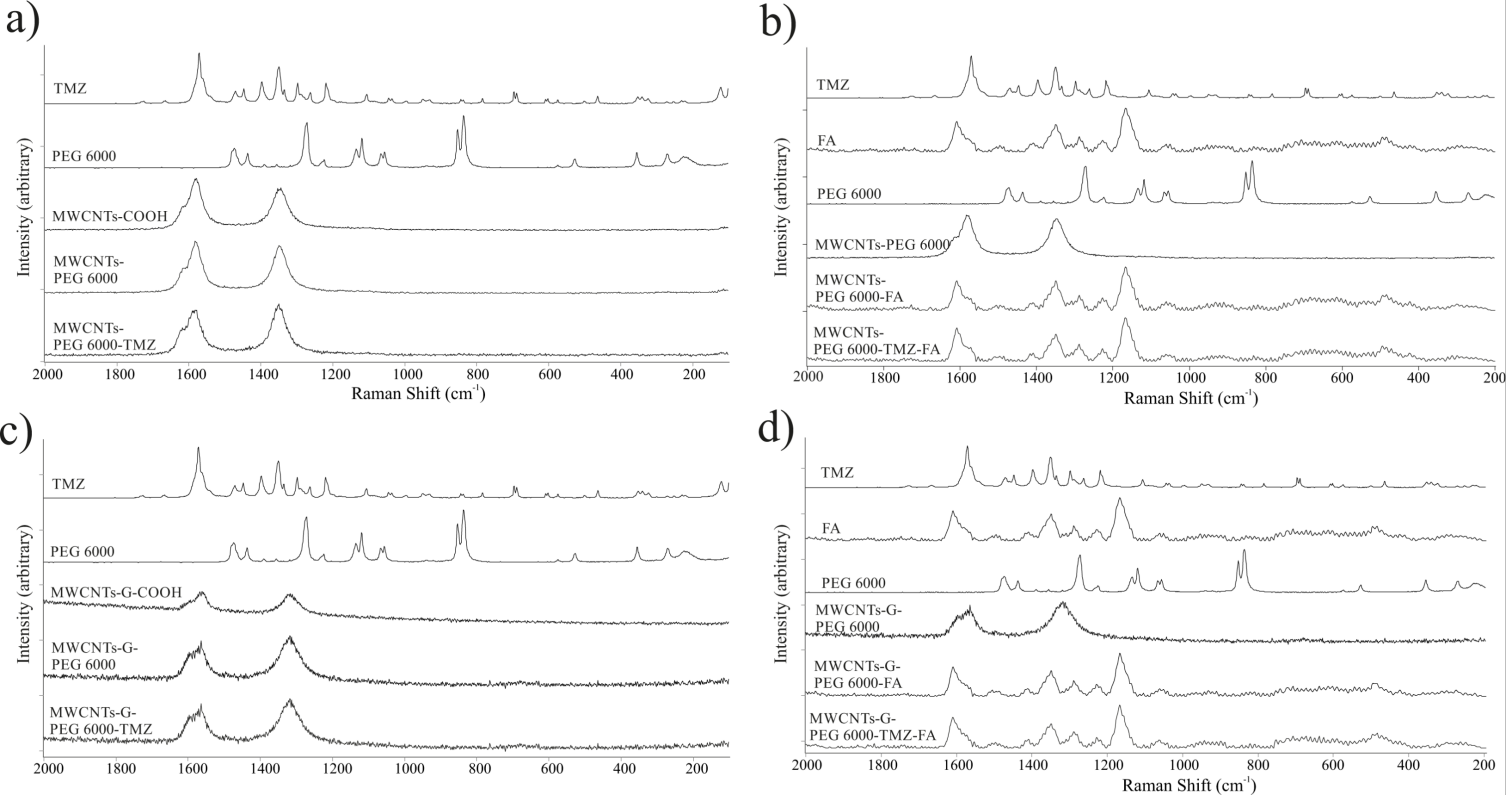
Raman spectra of individual components and blank and TMZ-loaded (a) MWCNTs functionalized with PEG, (b) MWCNTs functionalized with PEG and FA, (c) МWCNTs-G functionalized with PEG, and (d) MWCNTs-G functionalized with PEG and FA.

The Raman spectra of single- and dual-functionalized MWCNTs and MWCNTs-G, blank and TMZ-loaded, are presented in Figure 5. When analyzing these spectra, the following features were taken into consideration: the D (“disorder”) band, usually positioned around 1350 cm^−1^ and related to the degree of structural defects, deteriorations, and sp^3^-hybridization of the carbon atoms in the CNTs; the position and intensity of the G band related to the *E*_2g_ phonon mode of sp^2^-bonded carbon atoms in the 1600–1500 cm^−1^ region; the bands from the so-called low-frequency radial breathing mode (below 300 cm^−1^), sensitive to the diameter and chirality of the CNTs; the bands from the Raman modes that result from the vibration of all carbon atoms in the CNTs; and the 2D band occurring between 2600 and 2800 cm^−1^, which is sensitive to the number of graphene layers and their arrangement. The intensity ratio between the D and the G band was analyzed because of their significance for identifying the structural defects [60–62].

In the Raman spectra of MWCNT–COOH and MWCNTs-G-COOH (Figure 5a and Figure 5c, respectively), the intensity ratios of D and G band (*I*_G_/*I*_D_) were calculated as 0.59 and 0.65, respectively. The analogous calculation for modified MWCNTs-PEG6000 and MWCNTs-G-PEG6000 gave D/G intensity ratios of 0.64 and 0.75, respectively. The increase in *I*_D_/*I*_G_ ratios of the PEGylated CNs compared to the pristine ones indicates that the functionalization with PEG6000 induced a slight destructive effects on the surface of CNTs and confirmed the successful introduction of functional groups on their surfaces, that is, chemical modification of their outer layers [62]. The spectra of covalently functionalized MWCNTs-G with PEG6000 follow this behavior and are also accompanied by a broadening of the G band. The broadening and its intensity increase is indicative for a breakage of the graphene sheet symmetry, which is associated with the introduction of functionalities onto/into the structure of the MWCNTs-G hybrid.

A decrease in the intensity of all Raman bands was observed upon FA functionalization (Figure 5b,d). In addition, after dual functionalization and TMZ loading, further changes of the *I*_D_/*I*_G_ band ratio were observed. Namely, in the MWCNTs-based formulations, the values of 0.64 and 0.96 for MWCNTs-PEG6000 and MWCNTs-PEG6000-FA, respectively, increased to 1.17 and 0.98 for MWCNTs-PEG6000-TMZ and MWCNTs-PEG6000-FA-TMZ, respectively. These results point to increase and expansion of surface defects and successful functionalization. Functionalization by FA of MWCNTs-G-PEG6000 yielded an increase of the *I*_D_/*I*_G_ ratio from 0.75 to 0.85, while the corresponding lower values determined for MWCNTs-G-PEG-6000-TMZ and MWCNTs-G-PEG6000-FA-TMZ (0.60 and 0.79, respectively) point to a reverse trend during TMZ loading in the hybrid carrier, suggesting the formation of stable complexes between the drug and the carrier.

#### Thermogravimetric analysis

**Figure 6:**
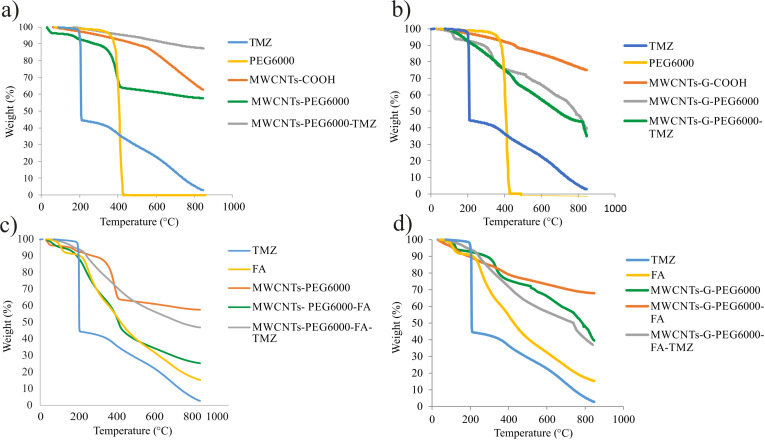
TGA diagrams of individual components and blank and TMZ-loaded (a) MWCNTs functionalized with PEG, (b) MWCNTs-G functionalized with PEG, (c) MWCNTs functionalized with PEG and FA, and (d) MWCNTs-G functionalized with PEG and FA.

The TGA findings are presented in Figure 6. As mentioned in our previous study [43] and also seen in the actual study (Figure 6a,b), the weight of pure PEG6000 decreased sharply with increasing temperature, showing almost 100% weight loss at ca. 400 °C. The same trend can be seen in the covalently functionalized MWCNTs-PEG6000 (Figure 6a,c), that is, 37% weight loss was observed at 400 °C (corresponding to the elimination of the functional groups with oxygen, i.e., H_2_O, CO_2_, and CO) and an additional 5% weight loss at 800 °C (42% weight loss in total). In the TGA diagram of covalently PEGylated MWCNTs-G (Figure 6b,d), a weight loss of 23% at 400 °C was observed, while at 800 °C, 39% of the weight remained (61% weight loss in total). For comparison, the TGA diagrams of non-covalently PEGylated MWCNTs and MWCNTs-G showed lower weight losses in total (29% and 52%, respectively) [43], pointing to a higher content of PEG6000 in the covalently PEGylated CNs.

In the thermogram of FA (Figure 6c,d), one can see a weight loss of 8% at 200 °C and up to 85% at 800 °C. After functionalization with FA, in the thermograms of both dual-functionalized CNs (Figure 6c,d), weight losses at two levels were observed, as in the thermogram of FA. Namely, in the thermograms of MWCNTs-PEG6000-FA (Figure 6c) and MWCNTs-G-PEG6000-FA (Figure 6d), 95% and 97% of the weight, respectively, remained at 200 °C, while at 800 °C, weight losses of 75% and 32% were observed, respectively, due to the decomposition of PEG and FA. After TMZ loading, in the thermograms of all formulations, non-significant or small differences in the weights were observed at 800 °C (Figure 6a–d) compared to the thermograms of corresponding formulations without TMZ (18%, 53%, 13%, and 59% for MWCNTs-PEG6000-TMZ, MWCNTs-PEG6000-FA-TMZ, MWCNTs-G-PEG6000-TMZ, and MWCNTs-G-PEG6000-FA-TMZ, respectively). This points to the formation of stable complexes and the absence of additional defects on the surface of CNs after TMZ loading.

#### X-ray powder diffraction

Several literature data are focused on the changes in structure and size of CNs after their exposure to gamma radiation. In one study [63], upon exposure of MWCNTs to gamma radiation and subsequent oxidation, the content of functional groups increased because of the increase in the effective surface for functionalization (or decrease in size) and the increase in number of defective sites on the MWCNTs created by the gamma photons. In addition, an exposure of SWCNTs to gamma radiation led to a significantly higher extent of functionalization with single-stranded DNA [64]. It was also shown that gamma radiation with low doses, 25 and 50 kGy, improved the graphite arrangement of MWCNTs; higher doses, 100 and 150 kGy, led to major structural deteriorations, while very high doses above 200 kGy distorted the structure [65]. There is also a great possibility that the changes in size and structure of CNs impact their physicochemical and biopharmaceutical properties (including solubility and permeability) [47].

**Figure 7:**
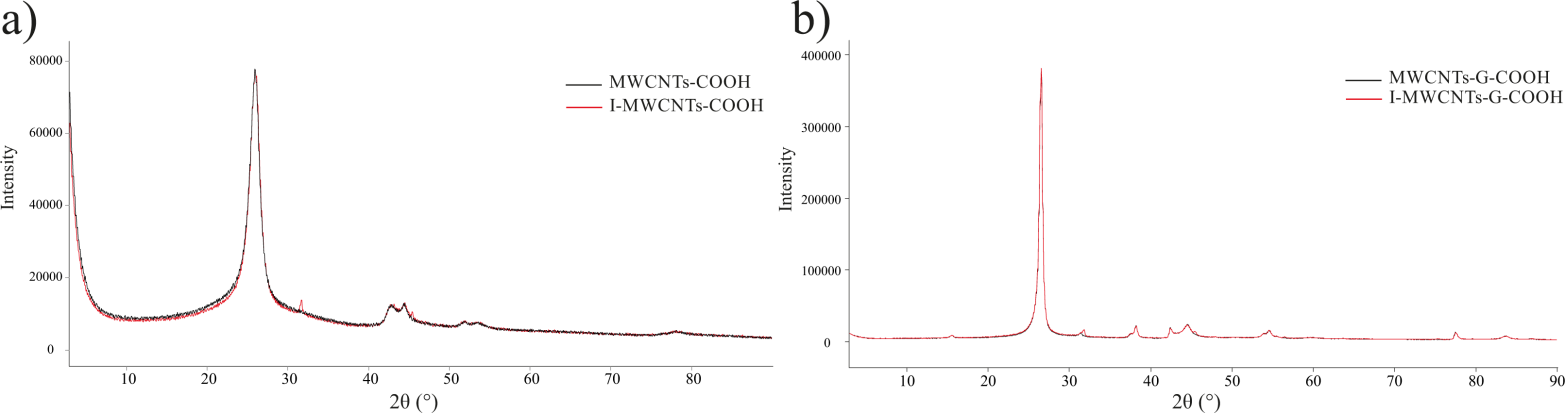
Comparison of XRPD diffractograms of non-irradiated and irradiated (a) MWCNTs-COOH and (b) MWCNTs-G-COOH.

The changes in the structure of the CNs after exposure to radiation were analyzed using XRPD. In the diffractograms of non-irradiated and gamma ray-irradiated CNs (Figure 7), two peaks important for the graphite arrangement of MWCNTs [66] were recorded at 2θ angles of 26° and 43°. For the irradiated MWCNTs-COOH, a rise of the intensity of the 26° peak was observed pointing to an increase of its crystallinity, as previously reported by Nie and coauthors [67]. Furthermore, an increase of the intensity of the peak at 43° was also observed in addition to an occurrence of two new peaks at 2θ angles of 31° and 45°. The peak at 31° can be assigned to the (002) interlayer spacing in nanotubes, while the other at 45° is related to changes in the crystallinity or arrangements in the nanotubes after irradiation. In the X-ray pattern of MWCNTs-G-COOH, the two characteristic peaks at 2θ angles of 26° and 43° were also present; however, the intensity of the peak at 26° decreased in the irradiated sample (pointing to structural imperfections), while the peak at 43° remained unchanged. No shifts of the peaks were observed in the X-ray patterns of both CNs pointing to non-significant changes in the structure of the CNs.

### Cytotoxic activity of temozolomide-loaded carbon nanostructures

A number of publications contain information on the potential mechanisms of CNs’ cytotoxicity, pointing to physical destruction, oxidative stress/ROS generation, DNA damage, cell autophagia and lysosomal membrane damage followed by mitochondrial dysfunction, pyroptosis followed by activation of inflammasomes, apoptosis via mitochondrial pathway and scavenger receptors, and necrosis [68–70]. For example, it has been shown that when single-wall CNTs (SWCNTs) enter into cells, they interact with the actin filaments, rearranging the actin, disrupt the mitochondrial function, and cause abnormalities in cell division; MWCNTs interact with tubulin and actin, causing cell apoptosis, which was confirmed in vitro [71–72] and in vivo [73–74]. In addition, genotoxic effects of CNTs have been identified by direct interaction with DNA [68]. For the G-family nanomaterials, induction of cell death, including apoptosis and necrosis was observed, with the involvement of toll-like receptor-, transforming growth factor β-, tumor necrosis factor α-, and mitogen-activated protein kinase-dependent pathways in the signaling pathway network and oxidative stress playing a crucial role in these pathways [75–76]. However, all publications emphasize that various factors may be involved in the induction of CNs’ cytotoxicity, These include physicochemical properties (e.g., diameter, length, size, type, and structure), solubilizing agents (e.g., PEG), modification of CNs (covalent or noncovalent functionalization), surface topology, aggregation behavior, oxidation status, and metal impurities (e.g., iron) in correlation with the cell type and concentration of CNs to which the cells are exposed.

**Figure 8:**
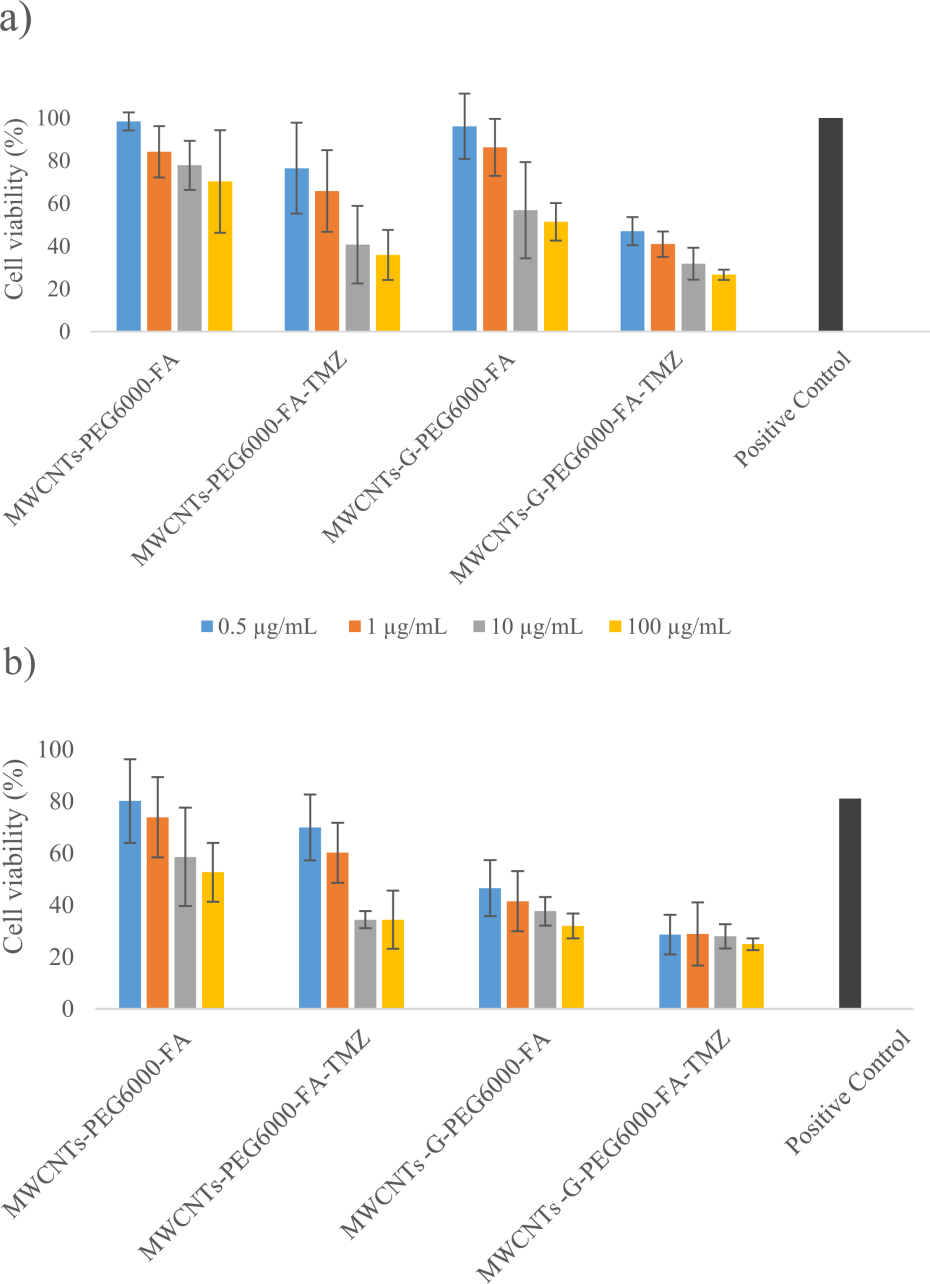
Viability of cell line U87MG after exposure to blank and TMZ-loaded dual-functionalized CNs. (a) Non-irradiated cells and (b) irradiated cells.

The results of the actual study in which the cytotoxicity of blank and TMZ-loaded dual-functionalized CNs was evaluated by the MTT test are presented in Figure 8. The results point to a concentration-dependent toxicity and a higher toxicity/potency when the cells are exposed to the hybrid CNs. Namely, the viability of the cells exposed to the blank MWCNTs-PEG6000-FA and MWCNTs-G-PEG6000-FA after 48 h of exposure ranged from around 98% (after exposure to the lowest concentration of 0.5 µg/mL) to around 70% and 51%, respectively, (after exposure to the highest tested concentration of 100 µg/mL). These data are in accordance with the findings in several studies in which cell uptake and cytotoxicity of different CNs in different cell lines were evaluated. In the study of Zhang et al. [77] in which cellular accumulation and cytotoxicity of NDs, MWCNTs, and GO in HeLa cells were compared, concentration- and time-dependent cytotoxicity was observed, with an uptake via non-specific mechanisms and rates in the following order: NDs > MWCNTs > GO. The cytotoxicity did not correlate with the uptake rate. For example, although uptake was highest, NDs showed the lowest toxicity, confirming that other factors contribute also to the CNs’ cytotoxicity. In the same study [77], it was shown that all CNs are internalized in the cytoplasmic organelles (lysosomes, mitochondria, and endoplasmic reticulum), that NDs and CNTs aggregate in big clusters into the cells, and that some of these aggregates are wrapped by extended cell synapses. Although NDs and CNTs were taken up by the cells to a greater extent, the cell membranes remained intact in contrast to the ruptured cell membranes observed after uptake of GO.

The mechanisms of MWCNTs-G internalization have not been specifically studied; however, the knowledge from studies of CNTs and graphene/GO can be extrapolated. Some of the previous publications show accumulation of CNTs in phagocytic cells without toxic effects [78–79], while another shows accumulation in microglia cells (BV2 glioma cell line) with uptake mechanisms that include energy-dependent (transcytosis) and/or independent mechanisms (needle like transfer through the cell membranes) [80]. Recently, it was shown that graphene enters into the cells generally via endocytic pathways; particles of several hundred nanometers or less are internalized by micropinocytosis, while the larger particles undergo phagocytosis or do not enter into the cells at all [81]. In the study of Huang et al. [82], GO was taken up by clathrin- (GO with diameter above 100 nm) and caveolin-mediated endocytosis (GO with diameter below 100 nm). In addition, accumulation in the glia cells due to the enhanced permeability and retention effect was observed. However, it is emphasized that surface modification, including modification of surface charge and particle size, can mediate the internalization in addition to the impact of interaction with the biological fluids (i.e., surface adsorption of molecules). In this respect, in the study of Dabrowski et al. [81], the impact of particle size of graphene on the efficacy of internalization was evaluated in normal (LL-24) and lung cancer cells (A549), whereby larger particles showed higher cell affinity. In another study, MWCNTs smaller than 8 nm were more toxic in 3T3 fibroblasts and bronchial epithelial cells compared with larger MWCNTs (20–30 nm and >50 nm, with the same length 0.5–2 µm); however, MWCNTs larger than 50 nm were more toxic than smaller ones for RAW264.7 macrophage cells [83]. It was also shown that long MWCNTs (20 µm) were cytotoxic to macrophages unlike short ones (0.6 µm) [84], that longer ones (825 nm) induced more intensive inflammation than shorter ones (220 nm) [85], and that longer CNTs had more significant biological effects such as cell death, ROS generation, and expression of macrophage inflammatory protein-1 alpha [86].

Considering the type of CNs, in one study [87], SWCNTs showed significantly higher toxicity than MWCNTs; however, both CNTs induced significant toxic effects at low concentrations. In a study of Wan et al. [88], it was shown that acid-functionalized SWCNTs and GO induced cell death, autophagosomal accumulation, and lysosome damage in the macrophages, but also that GO was more toxic despite the similarity in the chemical structure and surface functional groups, pointing to an influence of their different physical characteristics. In a study of Zhang et al. [89], pure 2D graphene showed lower concentration-dependent toxicity than highly purified SWCNTs in vitro (PC12 cells, similar to primary culture of fetal neurons) after 24 h of exposure; but in another study [90], the opposite was observed. Considering previously presented findings in the study of Zhang et al. [77] in which the cell uptake and cytotoxicity of NDs, MWCNTs, and GO were studied, the differences in cytotoxicity between the formulations based on MWCNTs and MWCNTs-G observed in the present study can be explained by the different effects on cell membranes during internalization. While the tubular shape of the CNTs provides membrane penetration whithout significantly affecting the integrity of the cell membrane, the flat surface of GO leads to more intense contact and rupture of the cell membranes. The same was also confirmed in a systematic review related to the cell toxicity and immunological effects of CNs, in which the toxicity of CNs followed the order: fullerenes < carbon black nanoparticles < MWCNTs < SWCNTs < graphene [69].

Regarding the surface modification, findings from all studies indicate that chemically modified CNs are highly water soluble and that their toxicity depends on the degree of functionalization. Considering the specific targeting ligands used for dual functionalization of the CNs in the present study, aside from their effects on the solubility of CNs, one cannot exclude their molecular interactions with the GBM cells and a contribution to the cytotoxicity of the formulations. Despite the evidence for excellent biocompatibility of PEG, there are papers demonstrating that PEG (depending on the molecular weight) may show selective dose- and time-dependent cytostatic effects on the proliferation of cancer cells by blocking the cell cycle in the G0/G1 phase and increasing the osmotic pressure [91–92]. In addition, in a paper of Xu et al. [93], dynamic biological interactions between PEG and cells on the molecular level were clarified, explaining both the inhibitory effect of PEG on cell growth and its biocompatibility by inducing metabolism modulations and survival autophagy through creating an intracellular hypoxic environment. Although there is no consensus on whether the role of folate in cancer cells is protective or harmful, a cytotoxic effect was also observed when FA was applied to breast cancer cells through induced oxidative stress as the driver of cytotoxicity, cell cycle arrest in the S and/or G1/G0 phases, and significantly increased apoptosis [94–95]. The mechanisms behind the cancer protection and potential carcinogenic effect of FA are in detail explained in a recent review by Thabet et al. [96], emphasizing its role in DNA synthesis and repair mechanisms, gene expression, protection from oxidative stress, homocysteine regulation, collagen synthesis, and effect on enzymes, on one side, and the stimulation of nucleotide synthesis and inactivation of tumor suppressor genes in correlation with overexpression of folate receptors, on the other side.

Including the differences in particle size of the CNs in the actual study (Table 1), that is, the slightly higher value for *d*_50_ of the dual-functionalized hybrid CN (374 vs 270 nm), similar values for the surface charge (−38 vs −33 mV), and the smaller length of the hybrid CN (less than 10 vs 10–30 µm, data from the producers), one can say that the higher fraction of flat surfaces in the hybrid CN, that is, the more intense contact with the cells and the length of the hybrid CN are dominant factors for its higher cytotoxicity.

After TMZ loading, the cytotoxicity of the formulations increased, showing a concentration dependence, with a preserved trend for higher cytotoxicity of the hybrid CN. In the tested concentration range (0.5 to 100 µg/mL), the viability of the cells exposed to MWCNTs-PEG6000-FA-TMZ ranged from around 76% to 36%, respectively, and from around 47% to around 27%, respectively, for the cells exposed to MWCNTs-G-PEG6000-FA-TMZ (Figure 8a).

After exposure of the cell line accordingly pre-treated with the blank or TMZ-loaded dual-functionalized CNs to irradiation, the viability of the cells additionally decreased compared to the viability of cells not exposed to irradiation (Figure 8b). The formulations based on the hybrid CN (MWCNTs-G) showed again higher (concentration-dependent) cytotoxicity in the tested concentration range of 0.5–100 µg/mL (concentration range of TMZ 0.00026–0.065 µM), with a viability of the cells after 48 h ranging from around 47% to around 32%, respectively, for MWCNTs-G-PEG-FA and from around 29% to around 25%, respectively, for MWCNTs-G-PEG6000-FA-TMZ. For comparison, the viability of the cells treated with MWCNTs-PEG6000-FA and MWCNTs-PEG6000-FA-TMZ in the same concentration range of CNs (concentration range of TMZ 0.00029–0.057 µM) and time interval ranged from around 80% to 53% and from 70% to 34%, respectively, while the viability of the control sample of cells exposed to irradiation only was around 81%. These data confirm the higher toxicity of the hybrid CN and point not only to the radiosensitizing properties of TMZ (showed in vitro [32,97] and in vivo [98]), but also to the radiosensitizing properties of the dual-functionalized CNs, especially the one based on the hybrid carrier. The overall data suggest that the treatment of GBM with the prepared TMZ-loaded CNs in combination with RT has the potential to yield significant toxicity and, thus, benefits in clinical settings.

The results obtained by MTT assay were also confirmed by measuring the activity of the released lactate dehydrogenase (LDH, a marker of membrane integrity) from the treated cells [99]. In the media of cells exposed to two concentrations (10 and 100 µg/mL) of the hybrid CN without TMZ (MWCNTs-G-PEG6000-FA), the activity of the released LDH was higher (249 and 255 U/L, respectively) compared to the activity of the LDH released in the media of cells treated with the same concentrations of MWCNTs-PEG6000-FA of 173 and 198 U/L, respectively. Higher activity was also observed in the media of the cells treated with the TMZ-loaded CNs compared to the unloaded ones, with values of 356 and 453 U/L in the media of the cells treated with 10 and 100 µg/mL MWCNTs-PEG6000-FA-TMZ, respectively, and 485 and 569 U/L, respectively, in the media of the cells treated with the same concentrations of MWCNTs-G-PEG6000-FA-TMZ.

Generally, the higher toxicity of the formulations after exposure to irradiation, aside from the synergistic activity with the gamma radiation, can be attributed to the changes in the structure and physicochemical and biopharmaceutical properties of the formulations. It is well known that gamma radiation generates ROS with subsequent oxidative damage of cell membranes, disruption of cell–cell tight junctions, and increase in cell permeability and apoptotic cell death [100]. However, there is a great probability in the actual study that the high penetrability of gamma rays led to a decrease in particle size, shifted their surface charge towards more positive values, and induced faster TMZ release (as observed when the formulations were directly exposed to irradiation with the same dose rate as in the cell studies, Table 1 and Figure 2c). Considering the literature data according to which glioma cells have a resting membrane potential during depolarization of around −20 to −40 mV [101], there is also a possibility that the lower size and the more positive values of the zeta potential of the CNs led to more efficient cell uptake and, thus, higher cytotoxicity when they were combined with RT.

One cannot clearly determine how the PEGylation and coupling with FA contributed to the overall radiosensitizing effect of the CNs. Several studies point to enhanced radiosensitizing properties of (drugs loaded in) PEGylated or FA-conjugated NPs when compared to pure NPs, mostly by a higher intracellular accumulation of radiosensitizer, leading to a higher rate of apoptotic cell death [102–105]. For example, in a study of Zhao et al. [102], significantly higher amounts of silver NPs were found in C6 glioma cells in vitro when they were PEGylated, showing higher sensitization enhancement compared to non-PEGylated NPs. The ratio was even higher when the PEGylated NPs were additionally functionalized with a guanine-rich DNA aptamer (As1411) with high binding affinity to a protein (nucleolin) overexpressed on the plasma membrane of C6 cancer cells. PEGylated gold NPs also showed greater potential as radiosensitizers in comparison to non-PEGylated ones in two GBM cell lines (U-87 MG and U-251 MG), pointing out that PEGylation improved the radiobiological efficacy, with induced double-strand breakage as an underlying mechanism, greater boost in RT immunogenicity, and with radiosensitization correlating with a greater upregulation of inflammatory cytokines [103]. Therefore, although the relative contribution of enhanced permeability and retention effect in passive accumulation within the tumor interstitium and active targeting to the folate receptors of GBM cells was not specifically analyzed in the present study, one cannot exclude the benefit of dual functionalization in selective and effective accumulation of the formulations in the tumor cells and enhancement of radiosensitizing effects, considering that they are directly related to the amount of intracellular radiosensitizers.

## Conclusion

In the actual study, covalently single- and dual-functionalized and TMZ-loaded CNs were prepared with physicochemical and biopharmaceutical properties suitable for crossing the BBTB, targeting the brain cancer cells, and controlling drug release. All TMZ-loaded formulations showed biphasic release profiles with sustained release of the drug over an extended duration. With suitable techniques for physicochemical characterization, the successful PEGylation, functionalization with FA, and loading with TMZ were confirmed; thus, the essential preconditions for extended circulation time, BBTB crossing, and internalization in brain cancer cells were fulfilled. The formulations of the covalently PEGylated hybrid CN (MWCNTs-G), with and without TMZ, compared to the corresponding formulations based on MWCNTs were characterized by similar TMZ content, higher mean particle size, similar surface charge, and faster TMZ release. These are related to differences in their structure, that is, higher content of surface-bound TMZ on the flat monolayer of graphene in the hybrid nanocarrier vs higher content of entrapped TMZ in the tubes of the MWCNTs carrier. After functionalization with FA, formulations with slightly lower content of TMZ, higher mean particle size, more negative surface charge, and with a similar TMZ release pattern were obtained, that is, dual-functionalized CNs with physicochemical and biopharmaceutical properties suitable for binding to folate receptors and uptake into GBM cells. Irradiation of the dual-functionalized CNs with a standard clinical gamma ray dose for patients with GBM led to certain changes in the crystallinity or arrangements of the CNs, accompanied by a decrease in mean particle size, shift of surface charge towards more positive values (although still negative ones), and faster TMZ release. Based on the viability and LDH activity of GBM cells, these formulations, whether blank or TMZ-loaded, showed significant concentration-dependent toxicity in the GBM cell line, with the formulations of the hybrid nanocarrier being more potent. This can be explained by a higher penetrability of the longer hybrid CN and a higher fraction of planar structures and, therefore, more intense contact with the cell membranes. After TMZ loading, the cytotoxicity of the corresponding formulations increased, preserving the trend for higher cytotoxicity of the hybrid NC and pointing to a synergistic effect between the CNs and TMZ. When GBM cells were irradiated after exposure to dual-functionalized blank and TMZ-loaded CNs, their viability additionally decreased. This can be attributed to the changes in the physicochemical and biopharmaceutical properties of the CNs and in the cell microenvironment and cell membranes, which leads to a higher cell uptake rate. These radiosensitizing properties of the dual-functionalized CNs, especially of the hybrid, accompanied by the radiosensitizing properties of TMZ suggest that the combination treatment of GBM with these formulations and RT has potentially a significant benefit in clinical practice. Overall, the results in this study present a solid base for further in vitro and in vivo studies aimed to develop TMZ-loaded formulations with optimal benefit/risk ratio for treating GBM.

## Experimental

### Materials

As carriers of TMZ, oxidized MWCNTs (MWCNTs-COOH; purity > 95 wt %, number of walls > 2, length 10–30 µm, outer diameter 30–50 nm, -COOH content 0.7 wt %, zeta potential −38 mV, average size 136 ± 20 nm) were purchased from Sisco Research Laboratories Pvt, Ltd, India, while the hybrid MWCNTs-G (purity > 99 wt %, length < 10 µm, inner diameter < 30 nm, outer diameter 30–100 nm, wall thickness 1–2 nm, zeta potential −29 mV, 810 ± 42 nm average size) were purchased from Incubation Alliance, Inc., Japan. For oxidation of MWCNTs-G, 98% H_2_SO_4_ and 65% HNO_3_ were used, both purchased from CarloErba Reagents S.A.S., Italy. MWCNTs-COOH and MWCNTs-G (previously activated to MWCNTs-G-COOH) were covalently functionalized with PEG [HO–(CH_2_–CH_2_–O)*_n_*–OH)] of average *M*_w_ 5000–7000 g/mol, *M*_n_/*M*_w_ ≈ 1.1 (PEG6000), purchased from Merck Schuchardt OHG (Germany). For covalent PEGylation, thionyl chloride (SOCl_2_), dimethyl formamide (DMF) and benzene, purchased from Merck Schuchardt OHG (Germany), and anhydrous tetrahydrofuran (THF) from Sigma-Aldrich (USA) were used. As a ligand for folate receptors on GBM cells, FA was used, supplied from Fisher Scientific (Belgium). During the functionalization procedure, the following chemicals were used: dimethyl sulfoxide (DMSO) purchased from Merck Schuchardt OHG (Germany) and carbonyldiimidazole (CDI) from Sigma-Aldrich (USA). TMZ was supplied by Sigma-Aldrich (USA), Polysorbate^®^ 80 by Sigma-Aldrich, UK. The antitumor activity/cytotoxicity of functionalized CNs with and without TMZ was investigated in vitro on glioblastoma cell lines of human origin, Uppsala 87 Malignant Glioma (U87MG), purchased from CLS Cell Lines Service GmbH (Germany). The cells were preserved in a medium consisting of Eagle’s minimum essential medium (EMEM; CLC Cell Lines Service GmbH, Germany), fetal bovine serum (FBS; Capricorn Scientific GmbH, Germany), penicillin (10.000 U/mL; Biological Industries Israel Beit Haemek Ltd., Israel) and streptomycin (10 mg/mL; Biological Industries Israel Beit Haemek Ltd., Israel). Trypsinization of the cells was performed with a mixture of EDTA–trypsin, purchased from Sigma-Aldrich (USA). All chemicals were of analytical grade.

### Methods

#### Functionalization of carbon nanostructures

In the first step, the hybrid MWCNTs-G was oxidized by a modified procedure described in our previous paper [43], in which 1 g of MWCNTs-G was added to 400 mL of a mixture of 98% H_2_SO_4_ and 65% HNO_3_ (v/v = 3/1), initially immersed in an ultrasonic bath for 8 h, with subsequent vigorous stirring at 80 °C for another 8 h. The resulting product was separated by centrifugation (3000 rpm, Eppendorf, MiniSpin, Germany), and the sediment was thoroughly and repeatedly washed with deionized water until reaching neutral pH. Afterwards, the oxidized hybrid (MWCNTs-G-COOH) was dried in an air dryer at 50 °C (ST-01/02, Instrumentaria Zagreb, Croatia).

For covalent PEGylation, a procedure described by Abdel Salam and Burk was used [106]. First, from cut and activated MWCNTs-G (as MWCNTs-G-COOH) and commercially purchased MWCNTs-COOH, CNs with carbonyl chloride groups (MWCNTs-G-COCl and MWCNTs-COCl, accordingly) were formed by dispersing the parent material (10 mg) in 200 mL SOCl_2_ and 5 mL DMF and mixing at 70 °C for 24 h. The samples were collected by centrifugation at 12000 rpm for 15 min (Eppendorf, MiniSpin, Germany), washed with anhydrous THF and dried overnight under vacuum. In the next step, MWCNTs-COCl and MWCNTs-G-COCl (5 mg), respectively, and PEG6000 (10 mg) were mixed 40 h in 100 mL solution of benzene/THF (v/v = 3/1). Afterwards, the samples were collected by centrifugation (12000 rpm, 15 min; Eppendorf, MiniSpin, Germany), washed with deionized water, and dried overnight under vacuum.

Coupling with FA was carried out according to a procedure described by Vu-Quang et al. [107] with certain modifications. FA and CDI, in quantities calculated according to the theoretical stoichiometric ratio of the terminal groups were mixed overnight in 3 mL solution of DMSO to activate FA. The covalently PEGylated CNs (MWCNTs-PEG6000 and MWCNTs-G-PEG6000), 10 mg each, were dispersed in DMSO, and the dispersions were added to the solution of activated FA with subsequent stirring in dark for 24 h (350 rpm, Vario Mag Multipoint, USA). Afterwards, the dispersions were transferred in a previously hydrated dialysis membrane and washed with deionized water until complete removal of unbounded FA (which was confirmed with measuring the concentration of free FA by UV–vis spectrophotometry at λ_max_ = 256 and 365 nm; UV/VIS Perkin Elmer Lambda 16, Arizona, USA). The dual-functionalized CNs were separated by centrifugation (12000 rpm, 15 min; Eppendorf, MiniSpin, Germany) and dried overnight under vacuum.

#### Temozolomide loading of carbon nanostructures

To 10 mL acidified aqueous solution of ТMZ (1 mg/mL, pH 2), 30 mg of plain (MWCNTs-COOH and MWCNTs-G-COOH) and single- (MWCNTs-PEG6000, MWCNTs-G-PEG6000) and dual-functionalized CNs (MWCNTs-PEG6000-FA and MWCNTs-G-PEG6000-FA) were added, accordingly. The dispersions were sonicated for 2 h (Ultrawave Limited, Cardiff, UK) and then magnetically stirred for 3 days (250 rpm/min) at room temperature (VARIO MAG Multipoint, USA). Afterwards, the CNs were isolated by centrifugation (12000 rpm, 15 min; Eppendorf, MiniSpin, Germany), washed with double-distilled water and dried in an air dryer (ST-01/02, Instrumentaria Zagreb, Croatia) at 37 °С for 72 h [43].

#### Biopharmaceutical characterization of temozolomide-loaded carbon nanostructures

**Drug loading efficacy and content.** In all formulations with TMZ, the efficacy of loading was determined as described in Equation 1. The concentration of TMZ in the supernatant (non-encapsulated TMZ) was determined by UV–vis absorption spectroscopy at λ_max_ = 328 nm (UV/VIS Perkin Elmer Lambda 16, Arizona, USA).


[1] $$\begin{array}{l} \text{drug loading efficacy}\left(\%\right)=\\ \frac{\text{total TMZ}\left(\text{mg}\right)-\text{nonencapsulated TMZ}\left(\text{mg}\right)}{\text{total TMZ}\left(\text{mg}\right)}\times 100\% \end{array}$$

The content of TMZ in the CNs was determined using Equation 2.


[2] $$\begin{array}{l} \text{TMZ content}\left(\%\right)=\\ \frac{\text{encapsulated TMZ}\left(\text{mg}\right)}{\text{mass of loaded MWCNT formulation}\left(\text{mg}\right)}\times 100\% \end{array}$$

All data were averaged from five measurements at least.

**Size distribution and surface charge.** The hydrodynamic diameter (z-average), polydispersity index (PDI), and zeta potential of all blank and TMZ-loaded CNs (non-functionalized, single-, and dual-functionalized ones) were determined by dynamic light scattering (NanoZS-100, Malvern Instruments Ltd., Worcestershire, UK) in wet dispersions. The wet dispersions were prepared by dispersing the formulations (5–10 mg) in 0.0001 M phosphate-buffered saline (PBS) by Ultra-turrax (T25 basic, IKA Werke, Cardiff, UK) for 1 min (13500 rpm). Small aliquots of the resulting dispersions were transferred to the measurement cell. At least six measurements were done for each sample.

The same procedure was used to determine the size distribution and surface charge of dual-functionalized formulations with and without TMZ after their exposure to irradiation. Namely, dispersions of MWCNTs-PEG6000-FA, MWCNTs-G-PEG6000-FA, MWCNTs-PEG6000-FA-TMZ, and MWCNTs-G-PEG6000-FA-TMZ in 0.0001 M PBS were placed in 96-well plates and exposed to irradiation at a dose of 5 Gy, with 6 MV photons, and a dose rate of 600 MU/min, using the Varian Clinic iX linear accelerator at the University clinic for radiotherapy and oncology. Previously, the plates were scanned with a GE Discovery CT590RT scanner, the treatment plan was prepared using Varian Eclipse ver. 16.1 system, and the dose rate was calculated using the AAA calculating algorithm. Subsequently, aliquots of the resulting dispersions were transferred to the measurement cell. At least six measurements were done for each sample.

**Morphology.** The morphology of all blank and TMZ-loaded non-functionalized, single- (with PEG6000), and dual-functionalized (with PEG6000 and FA) CNs was visualized by scanning electron microscopy (SEM, FEI Quanta 200, acceleration voltage 30 kV with EDS Oxford Inca Energy 350, UK, equipped with a secondary electron detector).

**In vitro dissolution test.** The drug release from all TMZ-loaded non-functionalized (MWCNTs-TMZ and MWCNTs-G-TMZ) and functionalized CNs (MWCNTs-PEG6000-TMZ, MWCNTs-G-PEG6000-TMZ, MWCNTs-PEG6000-FA-TMZ, and MWCNTs-G-PEG6000-FA-TMZ) was measured in vitro, in a procedure previously described [43], using a dialysis bag diffusion technique (dialysis membrane *M*_w_ cutoff 12000, Sigma-Aldrich, USA). Hermetically sealed formulations (≈30 mg) were suspended in 50 mL PBS pH 7.4. The entire experimental temperature was kept at 37 ± 0.5 °C, with continuous magnetic stirring at 100 rpm. At selected time intervals, up to complete TMZ release, the drug sample was removed from the receptor compartment with the replacement of the same medium. The concentration of released TMZ was quantified by measuring the concentration of the formed active hydrolytic product MTIC at pH 7.4 and 37 °C, using UV–vis absorption spectroscopy (λ_max_ = 255 nm, UV/VIS Perkin Elmer Lambda 16, Arizona, USA). All data were averaged from three measurements.

#### Physicochemical characterization of temozolomide-loaded carbon nanostructures

**Ultraviolet-visible spectroscopy.** UV–vis absorption spectra of all samples, including spectra of individual components and blank and TMZ-loaded non-functionalized and functionalized CNs were collected in a quartz cell with 1.0 cm path length using a UV/Vis Perkin Elmer Lambda 16 (Arizona, USA) spectrophotometer. The same apparatus was used to compare the spectra of TMZ released from irradiated and non-irradiated dual-functionalized formulations.

**Infrared spectroscopy.** IR measurements were performed on all individual components and blank and TMZ-loaded non-functionalized and functionalized formulations (2 mg of homogenized samples) using KBr pellets (200 mg dry potassium bromide per pellet). The characteristic absorption bands were recorded in the wavenumber range of 4000–400 cm^–1^ (Varian-660 FT-IR, Agilent Technologies, USA). In addition, spectrum of TMZ incorporated in dual-functionalized formulations before and after exposure to irradiation under the conditions described above was recorded and compared using an Agilent Cary 630 FTIR spectrometer with a ZnSe diamond ATR module (USA), in a wavenumber range of 4000–650 cm^−1^ and a spectral resolution adjusted to 4 cm^−1^ (2 mg homogenized dry sample). MicrolabPC software was used for data analysis.

**Raman spectroscopy.** Raman spectra of all individual components and blank and TMZ-loaded non-functionalized and functionalized formulations were collected on a LabRam 300 Infinity micro-Raman multichannel spectrometer (Horiba JobinYvon, Japan) using a He:Ne laser at 632.81 nm. An 1800 lines/mm grating monochromator was used to receive the backscattered radiation (180° configuration). Raman intensities were collected with a thermoelectrically cooled CCD. An Olympus LMPlanFL 50× objective (NA = 0.5) with a long working distance (10.6 mm) was used for magnification. A LaserCheckTM Handheld Power Meter (Coherent Scientific, Australia) was used to measure the laser power on the samples, which was 1.82 mW. The spectral collection time was set to 5 s, and the spectrum was averaged from five scans. For calibration purposes, the Rayleigh line at 0 cm^–1^ as well as the band of a Si standard centered at 520.5 cm^−1^ were used.

**Thermogravimetric analysis.** TGA on individual components and all blank and functionalized formulations with and without TMZ (7–10 mg per sample) were performed with a model Pyris 1 TGA (PerkinElmer, USA) under nitrogen atmosphere at a heating rate of 10 °C/min in a temperature range of 30–800 °C.

**X-ray powder diffraction.** Potential changes in the structure of the CNs (MWCNTs-COOH and MWCNTs-G-COOH) after exposure to irradiation under the conditions described above were analyzed using an X-ray diffractometer MiniFlex 600 C (Rigaku, Japan; Cu Kα radiation λ = 1.54178 Å). Diffraction data were collected in a 2θ range from 3° to 90° at a scanning rate of 4°/min, using a 1D detector D/teX Ultra2. The current intensity was 15 mA, and the voltage was set to 40 kV.

#### Cytotoxicity of temozolomide-loaded carbon nanostructures

**Cell culture.** Cytotoxic activity, including radiosensitizing effects of the blank and TMZ-loaded dual-functionalized CNs, was determined in vitro using the commonly used cell line U87MG for studying the effects on GBM. After seeding, the cell line was maintained in EMEM supplemented with 10% FBS, 10.000 U/mL penicillin, and 10 mg/mL streptomycin. It was cultured as a monolayer under sterile conditions in T-25 flasks at a temperature of 37 °C in a humidified atmosphere of 5% CO_2_. The cell medium was replaced every 2–3 days until reaching a confluence of 80%. After propagation of the cell culture, the cells were harvested by trypsinization with 0.1 mg/mL trypsin–EDTA solution in PBS, isolated by centrifugation (800 rpm, 5 min, BioSan LMC-4200R), resuspended in 5 mL EMEM, and used for further tests. All procedures were performed in accordance with the recommendations of the supplier.

**Irradiation and treatment of cell culture with temozolomide-loaded carbon nanostructures.** In order to evaluate the effects of blank and TMZ-loaded dual-functionalized CNs on the viability of the cells, 96-well plates containing monolayer cells in EMEM with 5 × 10^3^ cells/cm^2^ density were exposed to dispersions of MWCNTs-PEG6000-FA, MWCNTs-G-PEG6000-FA, MWCNTs-PEG6000-FA-TMZ, and MWCNTs-G-PEG6000-FA-TMZ in the cell culture medium at different concentrations (0.5, 1, 10, and 100 µg/mL) for 48 h. Afterwards, the viability of the cells was evaluated. As a positive control, the plates containing monolayer cells in EMEM, and as a negative control, cells maintained in Triton X-100 were used.

For evaluation of the radiosensitizing effects of the same formulations, the control cells (in EMEM and Triton X-100) and the cells treated with the same concentrations of the formulations in EMEM for 27 h were exposed to irradiation under the conditions described above. The cell viability after 48 h of incubation was determined.

**Viability of cell culture.** The viability of control cells and cells exposed to blank and TMZ-loaded dual-functionalized formulations (and irradiation) was determined by 3-[4,5-dimethylthiazol-2-yl]-2,5 diphenyl tetrazolium bromide (MTT) assay. In the predetermined time interval, the medium from the cells was discarded, and 20 µL MTT solution (5 mg/mL) was added into each well, and the 96-well plates were incubated for 4 h at 37 °C in 5% CO_2_ atmosphere. Afterwards, the supernatant was removed from the cells and the formed formazan crystals were dissolved in 150 µL DMSO. The optical density of the solution was measured at λ = 560 nm using a 2030 Multilabel Reader VictorTM X4 (Perkin Elmer, USA).

In addition to the MTT assay, lactate dehydrogenase (LDH) release assay was used for testing cell viability and/or cell membrane integrity. For this test, U87MG cells were treated with dispersions of MWCNTs-PEG6000-FA, MWCNTs-PEG6000-FA-TMZ, MWCNTs-G-PEG6000-FA, and MWCNTs-G-PEG6000-FA-TMZ in EMEM, 10 and 100 µg/mL each, for 48 h. Afterwards, in 200 µL of withdrawn supernatant, the activity of LDH was determined in accordance with the instructions given in the commercially available kit (Lactate dehydrogenase (LDH) ByoSystems S.A., Barcelona, Spain). As a positive and negative control, media from the cells in EMEM and Triton X-100, respectively, were used.

## Data Availability

Data generated and analyzed during this study is available from the corresponding author upon reasonable request.
